# Lines on a Skeleton

**Published:** 1909-07

**Authors:** 


### Lines on a Skeleton.

Behold this ruin! 'Twas a skull
Once of ethereal spirit full.
This narrow cell was Life's retreat,
This space was Thought's mysterious seat,
What beauteous visions filled this spot,
What dreams of pleasure long forgot,
Nor hope, nor joy, nor love, nor fear,
Have left one trace of record here.

Beneath this moldering canopy
Once shone the bright and busy eye,
But start not at the dismal void—
If social love that eye employed,
If with no lawless fire it gleamed,
But through the dews of kindness beamed,
That eye shall be forever bright
When stars and sun are sunk in night.

Within this hollow cavern hung
The ready, swift, and tuneful tongue;
If Falsehood's honey it disdained,
And when it could not praise was chained;
If bold in Virtue's cause it spoke,
Yet gentle concord never broke—
This silent tongue shall plead for thee
When Time unveils Eternity!

Say, did these fingers delve the mine?
Or with the envied rubies shine?
To hew the rock or wear a gem
Can little now avail to them.
But if the page of Truth they sought,
Or comfort to the mourner brought,
These hands a richer meed shall claim
Than all that wait on Wealth and Fame.

Avails it whether bare or shod
These feet the paths of duty trod?
If from the bowers of Ease they fled,
To seek Affliction's humble shed;
If Grandeur's guilty bribe they spurned,
And home to Virtue's cot returned—
These feet with angel wings shall vie,
And tread the palace of the sky!

ANONYMOUS.

